# The Valorisation of *Melissa officinalis* Distillation By-Products for the Production of Polyphenol-Rich Formulations

**DOI:** 10.3390/molecules29020377

**Published:** 2024-01-11

**Authors:** Eirini Stini, Dimitrios Tsimogiannis, Vassiliki Oreopoulou

**Affiliations:** 1 Laboratory of Food Chemistry and Technology, Department of Chemical Engineering, National Technical University of Athens, 5 Iroon Polytechniou, Zografou, 15780 Athens, Greece; eirstini@gmail.com (E.S.); ditsimog@chemeng.ntua.gr (D.T.); 2 NFA (Natural Food Additives), Laboratory of Natural Extracts Development, 6 Dios St., Tavros, 17778 Athens, Greece

**Keywords:** lemon balm, *Melissa officinalis*, by-products, extraction, rosmarinic acid, spray-drying encapsulation, freeze-drying encapsulation

## Abstract

Lemon balm (*Melissa officinalis*) is an aromatic and medicinal plant, rich in bioactive ingredients and with superior antioxidant activity. The essential oil of this plant is an expensive product, so the use of the by-products of the essential oil industry is particularly useful. The aim of this research was to process *Melissa officinalis* distillation by-products to develop a series of polyphenol-rich formulations. In the present research, lemon balm was distilled in a laboratory-scale distiller, and the recovered by-product was used for further successive extractions with acetone and water, using a fixed-bed semi-batch extractor. Acetone extract exhibited relatively poor results as far as yield, phenolic composition and antiradical activity are concerned. However, the aqueous extract presented high yield in both total phenolic content (i.e., 111 mg gallic acid equivalents (GAE)/g, on a dry herb basis (dw)), and anti-radical capacity (205 mg trolox equivalents (TE)/g dw). On a dried extract basis, the results were also impressive, with total phenols reaching 322 mg GAE/g dry extract and antiradical capacity at 593 mg TE/g dry extract. The phenolic components of the extract were identified and quantified by HPLC-DAD. Rosmarinic acid was the major component and amounted to 73.5 mg/g dry extract, while the total identified compounds were quantified at 165.9 mg/g dry extract. Finally, formulations with two different wall materials (gum arabic–maltodextrin and maltodextrin) and two different drying methods (spray-drying and freeze-drying) were applied and evaluated to assess their performance, yield, efficiency and shelf-life of total phenolic content and rosmarinic acid concentration. From the present investigation, it is concluded that after one year of storage, rosmarinic acid does not decrease significantly, while total phenolic content shows a similar decrease for all powders. According to the yield and efficiency of microencapsulation, maltodextrin alone was chosen as the wall material and freeze-drying as the preferred drying method.

## 1. Introduction

*Melissa officinalis*, commonly known as lemon balm, is a member of Lamiaceae family and it is considered a lemon-scented medicinal plant, rich in flavonoids, terpenoids and phenolic acids. The word Melissa comes from the Greek word (melissa), which means bee, since the plant is particularly attractive to bees [1]. The plant is native to Mediterranean territories, south-Central Europe, Central Asia and Iran.

The main triterpenes identified are oleanolic and ursolic acid [2]. Also, some of the flavonoids that have been identified are luteolin, apigenin, hesperidin, hesperetin, naringin, naringenin, catechin, epicatechin, rutin, quercetin, myricetin, quercitrin, rhamnocitrin and isoquercitrin [3,4,5]. Other identified compounds in lemon balm include phenolic acids (C_6_-C_1_), such as protocatechuic, and gallic acid, as well as the dimer of the latter (ellagic acid) and hydroxycinnamic acids (C_6_-C_3_) such as cinnamic, p-coumaric, caffeic and ferulic acid. A significant category of *M. officinalis* components belongs to the derivatives of caffeic acid, such as the simple esters of caffeic–quinic and caffeic-3,4-dihydroxyphenyl lactic acid, namely chlorogenic and rosmarinic acid, respectively. There are also reports that lemon balm contains rosmarinic acid condensation products with caffeic acid, such as lithospermic and salvianolic acid [6]. However, rosmarinic acid is considered the main bioactive constituent of lemon balm. It is worth noting that depending on the extraction method, the extraction solvent, the number of extraction steps, the size of the extraction particles and the temperature, the amount of the recovered bioactive compounds varies. 

Given that the main phenolic constituents of lemon balm are phenolic acids and flavonoids, many research groups have concluded that acetone and hexane are not good solvents for this material [7]. In contrast, ethanol, methanol and water are solvents with very satisfactory results. 

In particular, the optimisation of lemon balm extraction with water has been well studied, as water is the most economical and environmentally friendly solvent. Tulek et al. [8] concluded that the optimal conditions for the aqueous extraction of lemon balm are heating at 100 °C for 120 min. Palamutoglu et al. [9] observed the best recovery of phenolics, infusing 2 g lemon balm powder with 200 mL water at 95 °C for 240 s. They recovered 55 mg GAE/g dry weight of lemon balm powder (dw), and concerning rosmarinic acid, they recovered 16.4 mg/g dw. Fecka et al. [10] recovered 32.9 mg GAE/g dw and 21.9 mg/g dw rosmarinic acid by mixing 2 g of herb powder with 250 mL boiling water for 15 min. The infusions obtained by Barros et al. [11] (1 g powder mixed with 200 mL boiling water, for 5 min) recovered rosmarinic acid at a level of 55.68 mg/g dried extract. Silva et al. [12], who prepared infusions of lemon balm in a similar way (2 g powder mixed with 200 mL boiling water, for 5 min), obtained rosmarinic acid in the range of 34.41–41.71 mg/g dried extract. 

Aqueous ethanol and aqueous methanol mixtures were found to be more effective in the delivery of phenolic acids and rosmarinic acid in particular compared to water, while the acidification of the solutions further improved their activity [13,14]. Studies conducted by Angelov et al. [15], Sik et al. [14], Wang et al. [16], Kim et al. [17] and Duda et al. [18] examined the different ratios of ethanol and methanol solutions to optimise the extraction of phenolic compounds.

Lemon balm is known to have a great number of reported medicinal activities, including anxiolytic, antidepressant, neuroprotective, anti-anxiety, cardioprotective, cytotoxic, anti-inflammatory, hypoglycaemic, hypolipidemic, antioxidant, antimicrobial, antiviral, anticonvulsant, anti-angiogenesis and antiepileptic. According to Petrisor et al. [19], each substance has distinct medicinal activities, and they noted that rosmarinic acid is the main component responsible for the antioxidant and anti-inflammatory activity of lemon balm, and chlorogenic acid contributes to antidiabetic and antioxidant activity.

Nowadays, the utilisation of industry by-products, for the development of high-added-value products is of great importance and is one of the Sustainable Development Goals (SDGs) established by United Nations General Assembly. Lemon balm is an expensive herb, with low yields of essential oil. Therefore, the price of lemon balm essential oil is extremely high and its production is very limited. However, the potential utilisation of the distilled herbal mass of lemon balm (industrial by-product) as a source of polyphenols could both increase the sustainability of *M. officinalis* essential oil production and lead to the development of new antioxidant formulations for foods, supplements and cosmetics. The present work focuses initially on the extraction and analysis of the phenolic compounds of distilled lemon balm, in order to identify and quantify them in the plant. A novel approach of fixed-bed extraction with continuous solvent flow was applied, whilst water, at room temperature, was selected as an efficient and the most environmentally friendly solvent. The developed procedure could be easily upgraded to an industrial scale for the production of bioactive-rich extracts from lemon balm. The extracts were encapsulated in maltodextrin or in a mixture of maltodextrin with gum arabic, through spray-drying and freeze-drying, in order to obtain a series of powders that could be used as antioxidant additives in foods and cosmetics. Last but not least, the shelf-life of the product was examined over a period of one year.

## 2. Results

### 2.1. Identification and Quantification of Essential Oil Components

Dried lemon balm was distilled by water-steam distillation to obtain the essential oil and identify the volatile compounds. The distillation showed that the essential oil content of the plant was 0.06%. The literature data also report a low essential oil content of the plant, in the range of 0.02–0.3% [20]. GC-MS analysis of the oil was performed, and Table 1 shows the identified substances. 

The composition of lemon balm essential oil varies greatly depending on the origin of the plant, the different varieties and the geomorphological and weather conditions prevailing in the place where it is cultivated. Moradpour et al. [21] reported the presence of 1,3,8-*p*-menthatriene in the essential oil of lemon balm, eluted earlier than cis-citral, and followed by thymol and β-caryophyllene, in accordance with our results. In addition, δ-car-3-ene is reported in numerous studies [22]. On the contrary, alpha-caryophyllene has not been reported in the literature. From the results of Table 1, it can be seen that the main component of the examined lemon balm essential oil is neral (cis-citral), which is one of the main components of lemon balm essential oil according to the literature [23].

### 2.2. Identification and Quantification of Phenolic Components

#### 2.2.1. Recovery of Phenolic Compounds and Antiradical Activity

Τhe distillation residue was air-dried, powdered, and then, passed through a sieve with a mesh size of 600 µm. The resulting powder was subjected to two successive extractions with pure acetone, and then, with deionised water, in a fixed-bed extractor. Acetone was chosen as a non-polar solvent for the removal of lipophilic compounds from the plant, and deionised water was used for the recovery of water-soluble compounds. The solid-to-liquid ratio was kept constant at 1:10 for all extractions, as determined from preliminary tests performed and from the literature [24]. Increasing the amount of solvent results in more diluted extracts, a fact that might increase the cost of final drying of the extract. After obtaining the extracts, the total solids yields were determined for acetone and water extracts to be 28.7 mg/gdw (dw: dry plant weight) and 345 mg/gdw, respectively. The total phenolic content (TPC) of the extracts was determined using the Folin–Ciocalteu method, and the antiradical activity was tested via a DPPH radical assay.

The TPC recovered through the acetone extraction was 2.3 ± 0.2 mg GAE/gdw, while 111 ± 8 mg GAE/gdw was recovered through aqueous extraction. From these results, it was found that the aqueous extraction is much more efficient than the acetone extraction in terms of the phenolic content of the extracts. Furthermore, it should be noted that distilled lemon balm is a very rich source of phenolic compounds, since 11.1% of the initial herbal mass correspond to phenolics. Similarly, the antiradical capacity of the aqueous extract was much higher than that of the acetone extract; specifically, 205 ± 5 mg TE/gdw was found for the aqueous extract and 2.18 ± 0.06 mg TE/gdw for the acetone extract, respectively. From these results, it can be seen that the majority of the phenolic components and antioxidants of the distilled dry lemon balm are water soluble.

With regard to the extract consistency, the acetone extract had a TPC content equal to 81 ± 5 mg GAE/g dry extract and an antiradical capacity of 76.06 ± 0.05 mg TE/g dry extract., while the aqueous extract had a TPC content of 322 ± 20 mg GAE/g dry extract and an antiradical capacity of 593 ± 0.4 mg TE/g dry extract, respectively. From the above results, it can be understood that the aqueous extract is about 4 times richer in phenolic constituents than the acetone extract. Also, in terms of antiradical capacity, the aqueous extract is about 7.5 times more potent than that of the acetone extract. 

A total of 32.2% of the total solids of the water extract are quantified as phenolic compounds equivalent to gallic acid, a percentage that could classify the respective extract as very rich in phenolic compounds. A review performed by our research team concerning all the extracts obtained from Lamiaceae plants led to the conclusion that, on a dry extract basis, the highest value of total phenols ever recorder was for an ethanol extract from *Satureja thymbra*, which contained 289 mg GAE/g dry extract, published in 2017 [25]. In the case of lemon balm water extract, as mentioned above, the respective value was determined to be even higher at 322 mg GAE/g dry extract. Mambrouki et al. [7] reported 63 mg GAE/g dry extract concerning the ethanol extract of *M. officinalis*, while Papoti et al. [26] determined 200 mg GAE/g dry extract concerning a water infusion that was similar to the current water extract.

As mentioned above, the water extract presented antiradical activity equal to 593 mg TE/g dry extract. Trolox is a very potent radical scavenger, widely used as a reference compound for measuring the equivalent antiradical capacity of extracts. A different approach is the determination of EC50 values for pure compounds and extracts. EC50 represents the mass of dry extract (g) required for scavenging 50% of 1 kg DPPH, and consists of a direct measure of antiradical capacity. The activity of pure trolox was quantified at the level of 140 g/kg DPPH; thus, the EC50 of the water extract of lemon balm was calculated to be 237 g/kg DPPH. Pure potent antioxidants were quantified at 60 g/kg DPPH (quercetin) or even lower at 37 g/kg DPPH for rosmarinic acid by our research team. The respective EC50 of the extract from Satureja thymbra was determined to be 222 g/kg DPPH.

Based on the high efficiency of the aqueous extraction in terms of phenolic component recovery, an aqueous extraction of non-distilled dry lemon balm with deionised water, in a fixed bed extractor with the same operating conditions as the previous extractions, was performed. This extraction resulted in an extract with a TPC of 62 ± 5 mg GAE/gdw and an antiradical capacity of 77 ± 3 mg TE/gdw. The direct water extraction of the non-distilled plant led to quite poor recovery of phenolics. This could be the result of the lipid components that act as barrier to the effective penetration of water into the microstructure of the material. However, the sum of essential oil and the acetone-recovered material did not exceed 3% of the initial herbal mass, which is very low for such an impact on the water extraction. Therefore, it could be concluded that the better performance of water for the extraction of the polar phenolics occurred due to the structural changes in the microstructure caused by water–steam distillation. Indeed, steam causes swelling and reduces the cohesiveness of plant tissues, thus increasing the accessibility of the water solvent to the pores of the powdered plant. 

#### 2.2.2. Phenolic Compound Analysis

**Figure 1:**
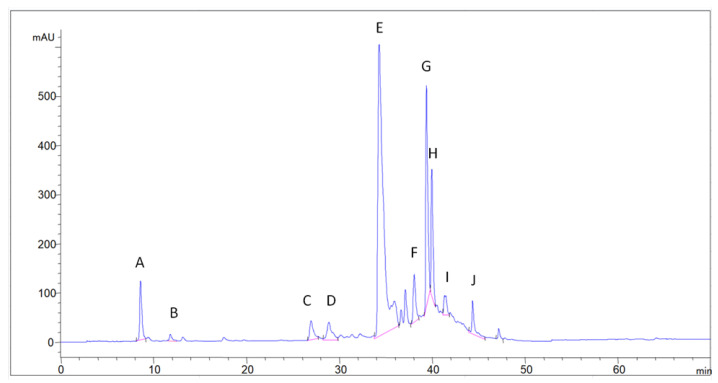
Chromatogram of aqueous extract of distilled and dried lemon balm at 360 nm.

The identification of compounds was carried out using internal standards, i.e., chlorogenic, neochlorogenic and rosmarinic acid, data from the literature on relative retention times and UV-Vis spectral data. The quantification was performed using calibration curves and the conversion of peak areas to concentration. A chromatogram of the aqueous extract of laboratory-distilled dry lemon balm, monitored at 360 nm, is presented in Figure 1.

Peaks A and B eluted at 8.6 min and 11.9 min, respectively. The spectra of A and B are shown in Figure 1 with the peaks’ absorption maxima. Based on the literature, lemon balm contains chlorogenic acid [19,27,28]. Siahpoush et al. [29] presented the spectrum of chlorogenic acid, which is identical to the respective spectra of peaks A and B. Furthermore, according to Szopa et al. [30], neochlorogenic acid elutes faster than chlorogenic acid from the reverse-phase column. Therefore, it is concluded that peak A corresponds to neochlorogenic acid and peak B to chlorogenic acid. Solutions of the two standard compounds were spiked as internal standards, confirming peaks A and B to be neochlorogenic and chlorogenic acids.

As described above, the main phenolic acid of lemon balm is rosmarinic acid, and in the present chromatogram, this compound eluted at 34.3 min (peak E) and was identified with the use of an internal standard solution of rosmarinic acid. 

Peaks C, D, F, H, I, J presented similar spectra to rosmarinic acid, and they were characterised as higher derivatives of caffeic acid and quantified as rosmarinic acid equivalents. Barros et al. [11], Miron et al. [31] as well as Carocho et al. [32] identified salvianolic acid A, lithospermic acid A and their derivatives at relative retention times higher than rosmarinic acid. In the current paper, peaks F, H, I and J match the respective criteria (spectra, and longer retention times than rosmarinic acid), and therefore, could be attributed to these compounds, which consist of more complex derivatives of caffeic acid. Concerning Peaks C and D, there is no clear evidence from the literature data, but possible candidates could be caftaric acid (ester of caffeic and tartaric acid) (C) and some caftaric acid hexoside (D). Caffeic acid was detected at 17.5 min, but only in trace amounts.

**Figure 2:**
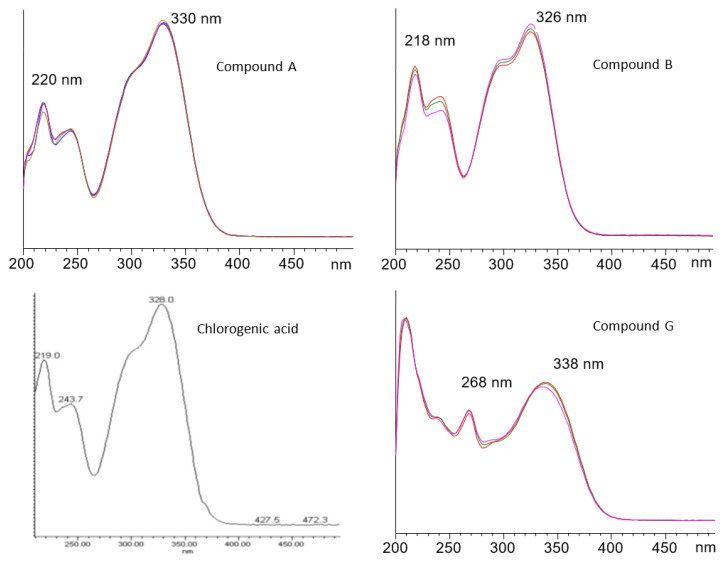
The characteristic UV spectra of compounds A, B and G as well as the respective spectrum of chlorogenic acid reported by Siahpoush et al. [29].

Peak G eluted at 39.4 min and presented a flavonoid-type UV-Vis spectrum, and was especially compatible with the subclass of flavones. The compound eluted in a short time after rosmarinic acid, and the UV-Vis spectrum obtained is presented in Figure 2. Barros et al. [11] and Miron et al. [31] both reported only one flavonoid eluting after rosmarinic acid for lemon balm extracts. They identified the compounds as luteolin-3′-O-glucuronide and luteolin-7-O-glucuronide, respectively. The UV spectral maxima of Miron et al. [30] for luteolin-7-O-glucuronide, i.e., 269 and 338 nm, are very close to the ones presented in Figure 2 (268, 338); therefore, the specific flavone could be attributed to the above glucuronide of luteolin (tentative identification). The compound was quantified in luteolin equivalents.

In the extract obtained, the total phenolic content was first determined, which was found to be equal to 111 mg GAE/gdw. Table 2 shows the results obtained for the quantification of the compounds of the aqueous extract.

According to the composition of the extract (Table 2), the main individual component, rosmarinic acid, amounted to 73.5 mg/g dry extract, when total phenols were quantified at 322 mg GAE/g dry extract. This roughly means that rosmarinic acid amounted to up to 23% of the total phenols and up to 52% of the total identified bioactives. It should be noted that, even in the ideal case whereby all individual phenols were identified and quantified, their sum could not match the TPC (Folin–Ciocalteu), since the two values were derived from two different methods. More specifically, in the case of TPC determination, phenolics are conventionally quantified as gallic acid equivalents. According to Berker et al. [33], who performed a whole series of calibration curves with both gallic acid and alternative standards, the ratio of molar absorption coefficients between the curve of gallic acid and rosmarinic acid is equal to 1:4. When transforming from molar to “per gram” absorption coefficients, the ratio becomes 1:1.9. This means that the TPC quantification of a solution of rosmarinic acid based on a gallic acid calibration curve produces a result that is 1.9 times higher than the respective one that is produced by the calibration curve of rosmarinic acid. Since the two standards present such a large difference in results, and our extract was composed mainly of rosmarinic acid and similar derivatives, the quantification of 322 mg GAE/g dry extract could be corrected to 169 mg RAE/g dry extract. This quantification is much closer to the sum of phenolic bioactives presented in Table 2 (165.9 mg GAE/g dry extract). Therefore, there is a minor difference, which could be attributed to the non-identified components of low concentration. 

### 2.3. Encapsulation of the Phenolic Extract

#### 2.3.1. Encapsulation Conditions

Maltodextrin and a mixture of gum arabic and maltodextrin (at a ratio of 1:4) were used for the microencapsulation of the bioactive components of the extracts. Maltodextrin is a polysaccharide, which is produced through the partial enzymatic hydrolysis of starch. Gum arabic is a secretion from the *Acacia senegal* and *Acacia seyal* trees. It is a complex branched heteropolysaccharide. Maltodextrin is soluble in water and readily dispersed in it, has low viscosity and a low content of simple sugars, is cheap and nutritious, provides good taste, protects against oxidation and its solutions are colourless [34]. The characteristics mentioned are some of the features that make maltodextrin a common coating material. It has been accepted that maltodextrin is the right alternative for gum arabic [35]. The combination of maltodextrin and gum arabic has been observed to increase the absorption of certain substances, such as flavonoids and their glycosides, compared to maltodextrin alone [36]. 

An aqueous extract of the distilled lemon balm was used for the microencapsulation experiments. The extraction was carried out 10 times in the laboratory fixed-bed reactor, and the water extracts were collected and combined to form a single water extract of 2.6 L volume. The aqueous extract contained 312 mg GAE/g dry extract and 65.3 mg rosmarinic acid/g dry extract. This extract was not used directly for the production of suspensions and encapsulation, but it was subjected to a concentration process, by using a rotary evaporator under reduced pressure and temperature (45 °C). The original 2.6 L volume of extract resulted in a concentrate of 1.0 L with total solids content of 41.3 g/L. The concentrated extract was separated in two equal parts, and suspensions were prepared, with two encapsulation carriers, maltodextrin and a mixture of gum arabic and maltodextrin (1:4). The core-to-wall ratio was adjusted to 1:4, where the terms core and wall stand for the solids of the extract and the carrier material, respectively. 

According to recent research results, spray-drying can be used for the encapsulation of bioactive compounds such as flavonoids to improve their physicochemical properties. Flavonoids without encapsulation are unstable under thermal changes, light, oxidation and oral administration, as they have low bioavailability via this route [37,38]. On the other hand, lyophilisation is a technique that finds wide application in thermosensitive components, such as polyphenols and essential oils [39,40]. 

The research of Tülek et al. [8] studied the encapsulation of an aqueous extract of lemon balm with maltodextrin as the encapsulation material with a core-to wall-ratio of 1:1 *w*/*w*. For the preparation of the powder, three different temperatures were investigated, 130 °C, 165 °C and 200 °C, with the condition of 165 °C presenting the best results. The antioxidant characteristic of the capsules did not show great variations at the different temperatures, but the increase in inlet air temperature improved the drying performance, the microencapsulation efficiency, the dry matter content of the capsules and the solubility of the capsules. Based on the above findings and laboratory experience, an inlet temperature of 160 °C and a 21% solid concentration in the feed was chosen.

#### 2.3.2. Encapsulation Yield and Efficiency

Table 3 presents the results of the encapsulation yield and encapsulation efficiency with either maltodextrin or the combination of maltodextrin and gum arabic, obtained via spray-drying and freeze-drying, respectively. Encapsulation yield expresses the percentage of polyphenols and rosmarinic acid transferred from the liquid extract to the dry powder through spray-drying and freeze-drying. Encapsulation efficiency measures the proportion of the previously transferred chemicals that are entirely contained inside the microcapsules, not just on the surface. Maximum (100%) yield and efficiency were defined as a hypothetical situation in which all of the extract’s polyphenols and rosmarinic acid were quantitatively recovered through spray-drying and freeze-drying and contained alone inside the dry powder’s microcapsules.

From the above results, it can be concluded that between the two selected microencapsulation wall materials and the two selected drying methods, the microencapsulation yields of total phenols and rosmarinic acid are high and show no statistically significant differences. The higher yield of 100% indicates complete uptake of the rosmarinic acid, and the higher percentage may be due to experimental variations. In terms of efficiency, both wall material microencapsulation options have very good efficiency for both rosmarinic acid and total phenols. However, the microencapsulation efficiency of rosmarinic acid in the case of the freeze-drying method is higher than that of spray-drying. Apart from the yield and efficiency of the drying methods, the moisture contents of the powders were determined. A low moisture content of the powders is desirable, as there is a maximum moisture limit to ensure the microbial stability of foodstuffs. In all cases, the moisture content did not exceed 6%, which is within the reported limit [41].

#### 2.3.3. Shelf-Life of the Powders

**Figure 3:**
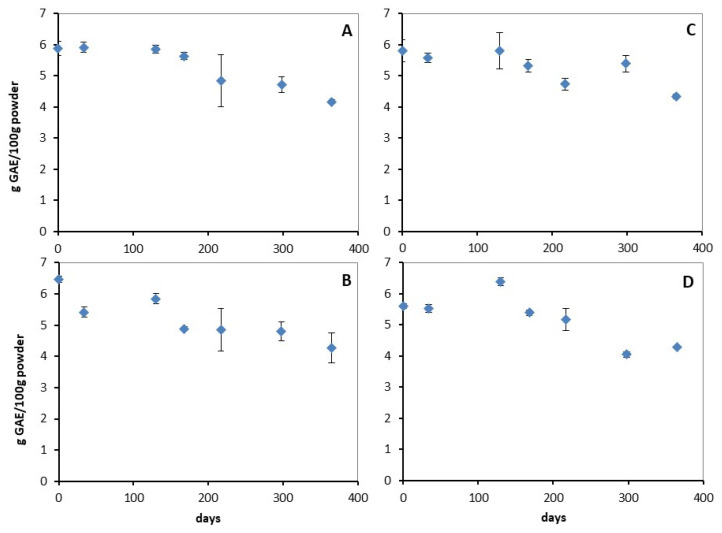
The evolution of TPC concerning the four powders (**A**–**D**) with encapsulated *M. officinalis* extract, stored in multilayer bags, at ambient temperature. (**A**) Maltodextrin–gum arabic—spray; (**B**) maltodextrin—spray; (**C**) maltodextrin–gum arabic—freeze; (**D**) maltodextrin—freeze.

In addition to the yield and efficiency of the microencapsulation of phenolic compounds and rosmarinic acid, the shelf-life of the powders was also studied in terms of total phenolic and rosmarinic acid content. Figure 3 presents the shelf-life of the powders in terms of their total phenolic content over a period of one year after production, stored in multilayer bags, under ambient temperature. 

The anova analysis showed that drying method and wall material has no statistically significant effect on the maintainability of the powders’ concentration of phenolic components. On the contrary, time has a statistically significant effect on shelf-life. In order to find the influence pattern, post hoc analysis with Duncans range test was conducted. The analysis revealed that there are five levels which are statistically different from each other. 

The levels are determined by the values of the samples among which there are no statistically significant differences. As a consequence, the first three samples determine the first level, and sample numbers four and five make level two. Level three consists of samples five and six, and finally, level four includes samples six and seven. The four levels are in descending order, followed by level five, which is determined by samples 2 and 4, which present no statistically significant difference between them.

It can therefore be seen that there is an almost linear decrease in the concentrations of the phenolic components of the powders with time. It is also important to mention that within the period of one year, the reduction observed in all powders is in the order of 28%.

In addition to the preservation of total phenolic compounds, the preservation of rosmarinic acid, which, as mentioned above, is the main phenolic component of the extract, was also analysed. As for the total phenolic components, Statistica was used, and the analysis performed used the three parameters of time, drying method of the samples and wall material. The anova analysis showed that drying method, wall material and time had no statistically significant effect on the retention of the concentration of rosmarinic acid in the powders.

Based on the above results, the shelf-life of total phenols and rosmarinic acid is not affected by the wall material and drying method. Therefore, the choice of the encapsulation wall material and encapsulation method is based on yield and efficiency. In order to find whether the yield and efficiency have a statistically significant difference with changes in the encapsulation wall material and encapsulation method, Statistica was used and a main effects anova was performed. The analysis showed that the encapsulation method and wall material have no statistically significant effect on the yield and efficiency of phenolic compounds. As regards the yield of rosmarinic acid, the encapsulation method and the carrier have no statistically significant effect on it, while in terms of efficiency, the encapsulation method has a statistically significant difference, with the freeze-drying method being better. Thus, maltodextrin is proposed as an encapsulation carrier, which is a more economical wall material, while freeze-drying is the preferred method.

## 3. Materials and Methods

### 3.1. Solvents and Reagents

Hexane for GC-MS analyses of essential oil was obtained from Fischer Scientific (Leicester, UK). Water and acetone for the extraction of dry distilled lemon balm were received from Fisher Scientific (Loughborough, UK) and Lach-ner (Neratovice, Czech Republic), respectively. Maltodextrin 18–20 DE, was used as carrier in the microencapsulation process and was purchased from Astron Chemicals (Attica, Greece), and gum arabic powder from Nexira (Rouen, France). For the analysis of the samples, Folin–Ciocalteu phenol reagent (2N) from Carlo Erba Reagents (Barcelona, Spain), sodium carbonate anhydrous from Penta Chemicals (Prague, Czech Republic), 2,2-diphenyl-1-picryl hydrazyl (DPPH) from Sigma-Aldrich (Steinheim, Germany) and gallic acid (98% *w*/*w*) obtained from Acros Organics (Fair Lawn, NJ, USA) were utilised. The standard compounds employed were rosmarinic acid from Sigma-Aldrich (Steinheim, Germany), luteolin, chlorogenic acid and neochlorogenic acid from Fluka (Buchs, Switzerland). For HPLC analyses of the samples, water, acetonitrile and methanol were used, which were obtained from Fischer Scientific (Leicester, UK), and trifluoroethanoic acid was obtained from Acros Organics (Fair Lawn, NJ, USA).

### 3.2. Plant Material

The plant material was supplied by Chiron Botanicals and included stems, flowers and leaves of dried *M. officinalis*. The plant was grown organically in the village of Drakeia, on Pelion Mountain (Greece), at an altitude of 600–1100 m.

### 3.3. Pretreatment Procedures

#### Grinding

The plant was ground in a household blender (Tefal Optimo, Rumilly, France) for about 1 min, and then, passed through a sieve with an opening mesh of 600 µm.

### 3.4. Water–Steam Distillation

Water–steam distillation is an intermediate technique between hydrodistillation (plant material is immersed into boiling water) and steam distillation (steam is supplied to the distiller by a steam generator). In water–steam distillation, the plant material is placed above boiling water, inside an additional perforated tank. This technique has also been described previously [25,42]. In the current study, water–steam distillation was applied for the removal of essential oil from lemon balm. The pilot-scale apparatus consisted of a 17 L copper distiller, into which 2.5 L of deionised water was placed. The tank was equipped with perforated mesh placed above the water surface. A total of 450 g of dry lemon balm was added without pressing the plant. Water was heated via electric resistance and the boiling rate was kept at a low level. The distillate flow rate was 4.6 mL/min. At the end of distillation, the residual plant was removed from the distiller and placed in a ventilated oven at 38 °C for 24 h for drying.

### 3.5. Extraction Procedure

#### Fixed-Bed Semi-Batch Extraction

A 65 mL vertical stainless steel fixed-bed extractor was filled with 25 g of powdered lemon balm and lightly filled with cotton. To introduce the solvent into the bed, a peristaltic pump (Millipore, MA, USA) was attached to the extractor’s input, and a flow rate of 3 mL/min was established. The extractor’s top allowed the solvent to exit after passing through the plant material and entering at the bottom. The study of Kaloudi et al. [43] presents the experimental setup diagrammatically. Acetone and deionised water were used as solvents, and a glass volumetric cylinder was positioned at the extractor’s outlet to collect the extract. Room temperature was used for the extraction. 

### 3.6. Encapsulation

#### 3.6.1. Preparation of Feed Mixture

Studies from the literature [8] combined with the laboratory experience were taken into consideration while choosing the spray-drying encapsulation settings. In relation to the feed mixture, a pre-treatment of the wall material was added to the distilled lemon balm extract. More specifically, 1.0 L of condensed extract with a total solids content of 41.3 g/L was produced from the initial 2.6 L of extract. The dissolution process was carried out gradually, with steady stirring and at a temperature of 30 °C to ensure complete homogenisation of the final mixture. The final feed mixture had a core:wall ratio of 1:4 and solids concentration of 21% *w*/*w*. The wall material was either maltodextrin (18–20 DE) or a combination of maltodextrin–gum arabic in a ratio of 4:1, *w*/*w*. The core material was assembled with the solids of the lemon balm extract.

#### 3.6.2. Spray-Drying Encapsulation

The spray-drying method followed in this research was based on well-established protocols [36]. Under constant stirring and at room temperature, each combination was delivered into the spray-dryer (Büchi B-191 Mini, Büchi Labortechnik AG, Flawil, Switzerland). The feed flow rate was set to 4 mL/min, the atomisation pressure to 5 bar and the air inlet temperature to 160 °C. In our experiments, the powder outlet temperature, a dependent variable in this procedure, ranged between 107 and 125 °C. On the same day, the created powder was gathered and submitted to further tests.

#### 3.6.3. Freeze-Drying Encapsulation

The selection of conditions and parameters concerning the freeze-drying process was based on previously published work [42]. The freeze-dryer used was the Alpha 1–4 LD plus, (CHRIST, Osterode am Harz, Germany). Seventy-five-millilitre portions of solutions were placed in small pre-weighed plastic containers. The samples remained overnight in the freezer. The frozen samples were placed in a drying chamber and the procedure of freeze-drying lasted for 5 days at −50 °C and 0.015 mbar pressure.

#### 3.6.4. Storage

The powders were packed immediately after preparation in multilayer bags [36], in the presence of air, and stored at room temperature. 

### 3.7. Analytical Procedures

#### 3.7.1. GC-MS Analyses

The study of the essential oil and the quantification of its components was achieved using GC-MS according to a well-described analytical protocol [43,44]. The system consisted of gas chromatograph 7890A (Agilent Technologies, Santa Clara, CA, USA) connected to a mass-selective detector 5975 C (Agilent Technologies, Santa Clara, CA, USA). The MS column used for the separation of the compounds was an HP-5 (30 m × 20 μm × 0.25 μm). A total of 4 μL of essential oil was dissolved in 10 mL of hexane (400 ppm), and 1 μL of the solution was injected into the chromatograph. The conditions of the analysis were as follows:

- Temperature: The initial temperature of the oven was 50 °C. After the injection, the temperature rose to 100 °C at a rate of 10 °C/min, and then, reached 220 °C at a rate of 15 °C/min.
- Carrier gas: helium, at a flow rate of 1 mL/min.
- Scanning range: mass-to-charge ratios, *m*/*z* = 40–400.

The identification of the compounds in the extract of lemon balm was carried out by comparing peak mass spectra with the data from NIST and Wiley mass spectral libraries. The composition of the essential oil in each of the compounds was obtained conventionally, as the % composition of the individual peak areas to the total peak area.

#### 3.7.2. Determination of Total Phenolic Content

The Folin–Ciocalteu reagent was used to calculate the samples’ total phenolic content (TPC). To measure the absorbance at 765 nm, a UV-Vis spectrometer (T90+, PG Instruments, Leicestershire, UK) was utilised. Each sample was subjected to two measurements, which were averaged. Gallic acid was used as a standard compound, and based on the respective calibration curve, the results were determined as gallic acid equivalents (GAE). The protocol has been described previously [45].

#### 3.7.3. Antiradical Capacity

By performing the DPPH radical assay with a UV-Vis instrument (T90+, UV-Vis spectrometer, PG Instruments, Leicestershire, UK), the antiradical capacity of the samples was determined. The measurements were based on the protocol described by Brand-Williams et al. [46]. A total of 0.1 mL of properly diluted sample was added to 3.9 mL of 6·10^−5^ M DPPH radical solution in methanol, and the absorbance at 515 nm was recorded after 30 min. Each sample had duplicate measurements, which were then averaged. Trolox was used as a standard compound, and according to the respective calibration curve, the results were determined as Trolox equivalents (TE).

#### 3.7.4. HPLC-DAD Analyses

The method used for the determination of phenolic acids and flavonoids of aqueous extracts of lemon balm has already been described [45], but in brief, was as follows. The mobile phase had a standard flow rate of 1 mL/min and comprised of a mixture of three solvents with a variable composition during the analysis. Solvent A was water, solvent B methanol and C was acetonitrile, and each one was acidified with 0.2% TFA. The initial composition of the solvent mixture was 90% A, 6% B and 4% C. Then, there was a linear gradient change to 85% A, 9% B and 6% C at 5 min; to 71% A, 17.4% B and 11.6% C at 30 min; and finally, to 0% A, 85% B and 15% C at 60 min, a composition that was maintained until the end of the analysis. For the static phase, a C18 reversed phase column, ZORBAX Eclipse XDB-C18, was used, with dimensions of 4.6 mm × 250 mm, and with a particle diameter of 5 μm and a pore size of 95 Å. The most representative acquisition with the DAD detector was at 360 nm. The validations of structures for chlorogenic, neochlorogenic and rosmarinic acid were carried out with the addition of the respective internal standards. The quantification of the specific phenolic acids the was based on external calibration curves of rosmarinic, chlorogenic and neochlorogenic acids. Similarly, the external calibration curve of luteolin was used for the quantification of luteolin-7-O-glucuronide.

#### 3.7.5. Encapsulation Yield and Efficiency

The encapsulation yield and efficiency of the powders produced by the spray- and freeze-dryers was performed according to the following procedure: 1 g of powder was dissolved in 5 mL of deionised water and stirred with a vortex for 30 s. The clear solution was subjected to HPLC-DAD and Folin–Ciocalteu analyses so as to determine the total content (TC) in terms of total rosmarinic acid content (HPLC) and total phenolic content (Folin–Ciocalteu). The yield (%) was determined as the ratio of the compound/s in the microcapsule (dry basis) to the compound/s in the feed (dry basis), multiplied by 100%. The surface content (SC) was determined by mixing 1 g of powder with 10 mL of ethanol:methanol solution (1:1, *v*/*v*) and stirring in a vortex for 1 min. The sample was filtered using a 0.45 μm pore filter, and the clear liquid was subjected to HPLC-DAD and Folin–Ciocalteu analyses. The efficiency was obtained according to the following general formula: microencapsulation efficiency = (TC − SC)/TC × 100%. When determining the efficiency in terms of total phenols, the TC and SC parameters were replaced by the respective results of Folin–Ciocalteu, and accordingly, with the results of HPLC analyses for the quantification of rosmarinic acid. 

### 3.8. Statistical Analysis

Statistical analysis was carried out using the software Statistica (Statistica 7, StatSoft, Hamburg, Germay). The analysis was performed in order to assess statistical differences in total phenolic content and rosmarinic acid concentration between the wall materials, drying methods and time. Also, the statistical analysis was used in order to examine significant differences in the yield and efficiency of microencapsulation between the wall materials and production methods employed. The results are expressed as mean values ± standard deviation. Main effects anova was performed for three parameters, time, drying method of the samples and wall materials. The differences between the group mean values were established at a 95% confidence interval (*p* < 0.05) using Duncan’s range test.

## 4. Conclusions

*Melissa officinalis* represents a superior source of water-soluble phenolic compounds with high antiradical capacity. The valorisation of the distillation by-product of the herb is definitely plausible, since distillation not only does not affect the quality characteristics of non-volatile compounds, but as proven in the current paper, the primary distillation of the herb could be beneficial to the yield following water extraction for the recovery of the phenolic antioxidants. The by-product of the laboratory-scale distillation of *Melissa officinalis* was further dried, powdered and subjected to water extraction, at room temperature, with fixed-bed extraction. The extraction presented very high yields, reaching 35% in terms of total recovered solids. The analyses of the extract revealed a very high content of total phenolics, 322 mg GAE/g dry extract, and high antiradical capacity, 593 mg TE/g dry extract. The identification and quantification of the aqueous extract showed that rosmarinic acid, quantified at 73.5 mg/g dry extract, was the major phenolic component of the extract, in agreement with the literature. 

The water extract was utilized for the production of a series of model microencapsulated powders as model ingredients for application in the food, supplement and cosmetic industries. The powders were obtained from microencapsulation using two different methods (freeze-drying, spray-drying) and with two different combinations of wall materials (maltodextrin, maltodextrin–gum arabic, 1:4). All of the produced powders had very high yields of total phenolics (89–100%) and rosmarinic acid (95–101%). Similar high results in terms of encapsulation efficiency were obtained, with 96.5–98.5% for phenolic components and 94–97.7% for rosmarinic acid. With the statistical analysis, it emerged that the yield of phenolic components in rosmarinic acid and the efficiency in phenolic components are not significantly affected by the drying method and the wall material, while in terms of the efficiency of rosmarinic acid, freeze-drying was at a higher level. The shelf-life of the powders (in terms of total phenolic content and rosmarinic acid) was studied over a period of one year. With the statistical processing carried out, it was found that a similar reduction (of approximately 28%) in the total phenolic components was observed for the powders obtained from both methods and from the two different combinations of wall materials. On the contrary, rosmarinic acid did not show a statistically significant decrease in any case.

## Figures and Tables

**Table 1:** The composition of essential oil recovered from lemon balm.

No.	Compound	RI	Ci (%)
1	δ-car-3-ene	1014	4.0
2	1,3,8-*p*-menthatriene	1125	28.8
3	cis-citral (neral)	1273	49.6
4	thymol	1296	0.2
5	(Ε)-β-caryophyllene	1437	16.6
6	alpha-caryophyllene	1489	0.8

**Table 2:** Quantification of phenolic compounds of aqueous extract of dry distilled lemon balm.

Compound	Concentration (mg/g) Dry Extract	Yield (mg/gdw)
Rosmarinic acid	73.5	25.3
Higher derivatives of caffeic acid	31	10.7
Luteolin-7-O-glucuronide	9.9	1.9
Chlorogenic acid	4.3	1.5
Neochlorogenic acid	47.2	16.3
Sum	165.9	55.7

**Table 3:** Encapsulation yield and encapsulation efficiency with maltodextrin and a 4:1 mixture of maltodextrin and gum arabic obtained via spray- and freeze-drying.

Total Phenolic Content	Yield (%)	Efficiency (%)
Spray-Drying/Maltodextrin	100 ± 1	96.8 ± 0.4
Spray-Drying/Gum Arabic–Maltodextrin	93 ± 4	96.5 ± 0.2
Freeze-Drying/Maltodextrin	89 ± 1	97.9 ± 0.1
Freeze-Drying/Gum Arabic–Maltodextrin	90 ± 4	98.5 ± 0.1
Rosmarinic acid content		
Spray-Drying/Maltodextrin	100.5 ± 0.1	94.0 ± 1.0
Spray-Drying/Gum Arabic–Maltodextrin	99.6 ± 0.9	94.5 ± 0.6
Freeze-Drying/Maltodextrin	101.2 ± 0.4	97.4 ± 0.1
Freeze-Drying/Gum Arabic–Maltodextrin	95 ± 3	97.7 ± 0.7

## Data Availability

Data are contained within the article.
